# UDP-glycosyltransferase confers anthranilic diamide resistance in *Bemisia tabaci*

**DOI:** 10.1016/j.jare.2025.10.077

**Published:** 2025-11-02

**Authors:** Huiwen Tan, Xichao Hu, Jinghao Hu, Wei Xu, Qianwen Wang, Xiaolan Liu, Lei Guo

**Affiliations:** a College of Plant Health and Medicine, Qingdao Agricultural University, Qingdao 266109, PR China; b Food Futures Institute, Murdoch University, Murdoch WA 6150, Australia; c Instrumental Analysis Center, Qingdao Agricultural University, Qingdao 266109, PR China

**Keywords:** Whitefly, Diamide insecticide, Metabolic resistance, Phase Ⅱ detoxification, Upregulation expression, UDP-glucose biosynthesis

## Abstract

**Introduction:**

UDP-glycosyltransferases (UGTs) are key Phase II detoxification enzymes in insects, playing a crucial role in resistance to a variety of insecticides, including diamides. Cyantraniliprole (CYA) is a systemic diamide insecticide with high toxicity against *Bemisia tabaci*, a major global agricultural pest transmitting over 200 plant viruses. While *B. tabaci* has developed medium-level resistance to CYA, the role of UGTs in this resistance remains poorly understood.

**Objectives:**

This study aimed to investigate the role of UGTs in *B. tabaci* resistance to CYA and further elucidate the underlying mechanisms.

**Methods:**

Synergism bioassays and enzymatic activity analyses were conducted to assess the contribution of UGTs to CYA resistance. RNA sequencing and RT-qPCR were employed to identify differentially expressed *BtUGT* genes associated with resistance. The functions of UGTs and the UDP-glucose (UDPG) biosynthesis pathway in CYA resistance were confirmed through RNA interference and transgenic *Drosophila melanogaster* lines. The metabolites of CYA in the resistant strain were identified using LC-MS/MS. A cross-resistance study to chlorantraniliprole was performed to further confirm the role of UGTs in the diamide resistance of *B. tabaci*.

**Results:**

Higher expression of UGTs represents a key metabolic resistance mechanism of *B. tabaci* to CYA. Specifically, overexpression of *BtUGT352B2* and *BtUGT352F1,* along with the UDP-glucose biosynthesis pathway, contributed significantly to resistance. Four glycoside metabolites of CYA were putatively identified in resistant whiteflies. Significant cross-resistance to chlorantraniliprole confirmed that the UGT-mediated metabolism plays a crucial role in anthranilic diamide resistance in *B. tabaci*.

**Conclusion:**

This research advances our understanding of UGT-mediated insecticide resistance mechanisms and provides valuable insights for the development of more sustainable pest management strategies to combat pesticide resistance.

## Highlight

• UDP-glycostransferases (UGTs) plays a key role in cyantraniliprole (CYA) resistance in *Bemisia tabaci*.
• Functional validation confirmed the roles of *BtUGT352B2* and *BtUGT352F1* in confering CYA-resistance.
• The UDP-glucose biosynthesis pathway is implicated in the CYA-resistance mechanism*.*
• Four glycoside metabolites of CYA were putatively identified in resistant pest.
• UGTs mediated the cross-resistance of *B. tabaci* to chlorantraniliprole.

## Graphical abstract

**Figure:**
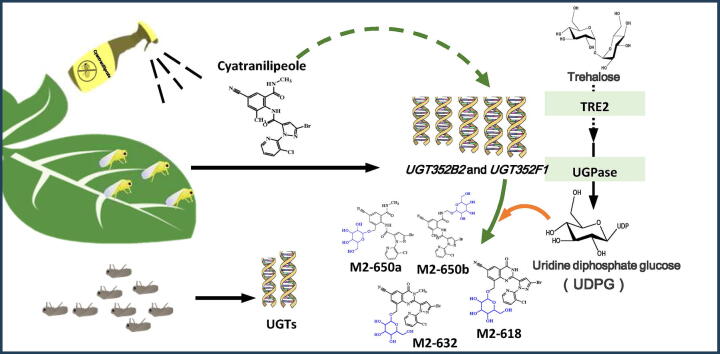

## Introduction

UDP-glycosyltransferases (UGTs) are a multifunctional superfamily of enzymes present in all living organisms [1]. UGTs catalyze the conjugation of lipophilic exogenous and endogenous compounds with sugars, producing hydrophilic glycosides that can be efficiently excreted, thereby protecting the internal systems from the detrimental effects of exogenous substances and maintaining internal homeostasis [2]. The glucose conjugation of lipophilic compounds by UGTs has been recognized as a key detoxification mechanism in insects against plant secondary metabolites [3]. Recent studies have revealed the pivotal role of glucose conjugation reactions catalyzed by insect UGTs in the detoxification metabolism of multiple insecticides [4,5].

Insect UGT enzymes primarily use UDP-glucose (UDPG) as a glycosyl donor, transferring the glucose moiety to the target substrate via a glycosylation reaction [6]. The known biosynthetic pathways of UDPG include three main routes, in which the glucose-6-phosphate metabolic pathway is the most predominant and ubiquitously present route for UDPG synthesis [7,8]. In insects, trehalose is hydrolyzed by trehalase (TRE) to glucose, which, following a series of metabolic steps, is ultimately converted to UDPG by the catalytic action of UDPG pyrophosphorylase (UGPase) [9,10]. Therefore, glucose-6-phosphate metabolic pathway provides the essential substrate (UDPG) for UGT-catalyzed glycosylation reactions in insects, facilitating the metabolism and excretion of both exogenous and endogenous compounds.

Diamide insecticides, following the success of neonicotinoids, have become the most promising insecticides on the market and play an essential role in controlling different types of pests [11]. For instance, chlorantraniliprole (CHL) currently ranks as the highest-grossing insecticide worldwide. Similarly, cyantraniliprole (CYA), as the first diamide insecticide to effectively target piercing-sucking pests, plays a pivotal role as a key alternative in combating resistance in hemipteran species [12]. However, repeated application of diamides has led to field control failure in various regions worldwide [13,14]. Previous studies have revealed that UGTs played a pivotal role in the metabolic resistance of pests to diamides in *Plutella xylostella* [15], *Chilo suppressalis* [16], and *Tuta absoluta* [17]. However, since Phase II detoxifying enzymes frequently cooperate with Phase I detoxifying enzymes, studies on the metabolites of diamide insecticides in resistant pests are limited. Nevertheless, this information is crucial for a comprehensive understanding of the metabolic processes of diamide insecticides and provides valuable insights into the degradation of these insecticides in non-target organisms and the environment.

The whitefly, *Bemisia tabaci*, is a major agricultural pest that can feed on over 400 plant species and transmit over 200 plant viruses, posing a grave threat to agricultural production [18]. Moreover, *B. tabaci* has developed moderate to high resistance to most insecticides due to its short generation cycle, broad host range, and rapid development of resistance [19]. Although CYA demonstrates remarkable efficacy against *B. tabaci* populations that have developed high resistance to other insecticide classes, some field populations in China have recently developed over 1000-fold resistance to CYA [20]. Although the involvement of *UGTs* in insecticide resistance has been detected in *B*. *tabaci* [21], its contribution to resistance against CYA remains poorly understood.

In the present study, the role of UGTs in CYA-resistance was confirmed in four resistant strains of *B*. *tabaci* using synergistic bioassays, UGT activity measurements, and kinetic parameter analysis. After identifying two upregulated UGT genes (*BtUGT352B2* and *BtUGT352F1*) using RNA sequencing (RNA-Seq) and reverse transcription quantitative real-time PCR (RT-qPCR), their roles in resistance were investigated using RNA interference (RNAi) and transgenic *Drosophila melanogaster* lines. The potential role of UDPG in CYA resistance was assessed by knocking down two key genes involved in the UDPG biosynthesis pathway. Glycoside metabolites of CYA in the resistant *B. tabaci* strain were identified using LC-MS/MS. Finally, the cross-resistance study to chlorantraniliprole further confirmed the involvement of UGT-mediated metabolism in the resistance of *B. tabaci* to diamide insecticides. These results enhance our understanding of the role of UGTs in resistance to cyantraniliprole and provide insights for the development of more sustainable pest management strategies.

## Methods and Materials

### *B. tabaci* strains

One susceptible strain (QS strain) and four resistant strains (QR, SG19, SG20, and WF20 strain) of *B. tabaci* were used in this study, in which the information of QS, QR and SG19 has been reported in our previous study [22]. The susceptible strain (QS strain) was collected in the cotton field from Jinan, Shandong Province in 2012. The QR strain, derived from the QS strain, has undergone selection with CYA in every generation since April 2018. The remaining three resistant strains, SG19, SG20, and WF20, were obtained from tomato fields in different locations and time periods: Shouguang, Shandong, in October 2018; Shouguang, Shandong, in June 2020; and Hanting, Shandong, in July 2020. They have all been subjected to CYA selection in every generation since December 2018, July 2020, and October 2020, respectively. Briefly, the adults of resistant strains were treated with CYA for 48 h, and the surviving adults were transferred to a cotton seedling without insecticide treatment to breed and produce the next generation. The screening concentration was determined based on the LC_50_ value derived from the bioassay results of each resistant strain, with the exposure time consistent with the bioassay duration. All strains were reared on cotton plant (*Gossypium hirsutum* L. var. “Lumian 28”) at 27 ± 1 °C, 60 ± 5 % relative humidity, and a 16:8 (L/D) photoperiod.

### Toxicity assessment of *B. tabaci*

A leaf-dip bioassay was used to evaluate the toxicity of CYA to *B. tabaci* adults. The technical grade CYA (94 %, FMC) was initially dissolved in dimethyl sulfoxide (DMSO) and subsequently diluted to five concentrations using deionized water with 0.5 ‰ Triton-X-100. The different concentrations of CYA could cause 10 %–90 % mortalities of *B. tabaci*. For the susceptible strain, the concentrations were set as 30 mg/L, 10 mg/L, 3.33 mg/L, 1.11 mg/L, and 0.37 mg/L. For the resistant strains, the concentrations were set as 200 mg/L, 100 mg/L, 33.3 mg/L, 11.1 mg/L, and 3.7 mg/L. The cotton leaf with a diameter of 2.5 cm was immersed in CYA solution for 10 s. After dried in air, the leaf disc was positioned with the abaxial side down on a bed of agar (1.0 %) within plug-sealed caps. Approximately 15 *B. tabaci* adults were transferred to the tubes and covered with the leaf disc inside caps. Mortality was assessed after 48 h. Three technical replicates were performed for each concentration and the control group (immersed in distilled water). The LC_50_, slopes, 95 % confidence limit (95 % CL) values, and chi-square values in bioassay results were calculated using PoloPlus 2.0 (LeOra Software, Petaluma, USA), with dose-mortality response analyzed by the Probit model and its linearity assessed using the Chi-square test.

To investigate the role of UGTs in CYA resistance, two UGT enzyme inhibitors, sulfinpyrazone (Sul, 98 %, Macklin, China) and 5-nitrouracil (5-Nul, 97 %, Yuanye, China), which are widely recognized as broad-spectrum UGT inhibitors in insects, were used as synergists in the bioassay [15,23,24]. These synergists were dissolved in acetone to prepare a stock solution at 5 × 10^4^ mg/L, which was subsequently diluted to 400 mg/L in various concentrations of CYA. Prior to the synergist bioassay, the effects of solvents and synergists on *B. tabaci* were assessed and determined to be negligible. Mortality rates for 400 mg/L SUL (mean ± SD: 2.4 ± 4.1 %), 400 mg/L 5-Nul (4.4 ± 3.8 %), 1 % DMSO (3.2 ± 3.7 %), and 1 % acetone (2.6 ± 3.5 %) in deionized water containing 0.05 % Triton X-100 were not significantly different from the control group (2.6 ± 3.7 %). The same leaf-dip method described earlier was used for these assays.

### Enzyme activity assay of UGTs

To measure UGT activities in different strains of *B. tabaci*, the catalytic ability of UGT to convert the substrate α-naphthol into α-naphthyl glucoside was utilized as the basis for the assay [25]. Approximately 40 *B. tabaci* adults were ground in 800 µL sodium phosphate buffer (0.05 mol/L, 7.8 pH), containing a mixture of NaH_2_PO_4_ and Na_2_HPO_4_ to maintain the desired ionic strength and pH. The homogenate was centrifuged at 4 °C, 12,000 g for 15 min, and the supernatant was utilized as the enzyme source. After 10 µL 250 mM MgCl_2_ and 10 µL 25 mM UDPG were added in a 220 µL enzyme solution in a well of a 96-well plate, 10 µL 200 µM α-naphthol was added last in the reaction. The fluorescence intensity was immediately measured at 335 nm wavelength with excitation at 287 nm wavelength in every minute for 15 min using microplate reader (Infinite 200 PRO, Tecan, Austria). UGT activity (pmol/min/mg protein) was calculated from the production of 1-naphthyl-glucopyranoside and normalized to protein content of the samples. The linearity of 1-naphthyl-glucopyranoside concentration was confirmed within the standard range of (15,625–2,000,000) pmol/L. The protein concentration of the samples was determined using the BCA assay by comparing their absorbance to a standard curve generated from BSA standards (0.125–4 mg/mL) through regression analysis. Three biological replicates in each strain and the three technical replicates were conducted.

To further explore the kinetic properties of UGT activities in different *B. tabaci* strains, a kinetic analysis was performed by varying the concentrations of the substrate α-naphthol in the reaction mixtures (final concentrations of α-naphthol were 160, 80, 40, 20, 10, 5, and 2.5 µM in different reactions). Over 15 min, fluorescence intensity was measured to track the progress of the enzymatic reaction. According to the Michaelis-Menten enzyme kinetic model, *K_m_* and *V_max_* were calculated by using GraphPad Prism 7.04 software.

### RNA sequencing

According to the enzyme assay, UGT activity in resistant strains was significantly higher than that of the susceptible strain. Therefore, differentially expressed *UGT* genes between the susceptible strain and four resistant strains were identified through high-throughput RNA-Seq. Briefly, at least 150 *B. tabaci* adults in each replicate sample and three replicates in each strain were collected. The total RNA was extracted using TRIzol agent (Thermo Fisher Scientific, CA, USA) and treated with DNase I (Thermo Fisher Scientific, CA, USA). The integrity of RNA was assessed using Agilent 2100 Bioanalyzer (Agilent Technologies, CA, USA). Samples with RNA integrity values of 7 or above were subjected to library preparation using the TruSeq Stranded mRNA LT Sample Prep Kit (Illumina, San Diego, USA) and sequenced on the Hiseq-2500 sequencing platform (Illumina, San Diego, USA). The raw reads containing poly-N sequences and low quality were trimmed using Trimmomatic software [26]. The filtered reads were aligned to the *B. tabaci* reference genome (NCBI_ASM185493v1) using HISAT 2 software [27]. Principal component analysis (PCA) was performed on gene count data, which was normalized using the rlog transformation to mitigate technical variation and nonlinear effects across samples. The normalized data were subjected to PCA for dimensionality reduction, extracting the principal components. Three-dimensional PCA visualizations were subsequently generated using the pca3D package. Fragments per kilobase of transcript per million mapped reads (FPKM) and read counts per thousand bases were calculated using Bowtie 2 [28] and eXpress software [29], respectively. Differential expression analysis was performed using the estimateSizeFactors function for data normalization and the nbinomTest function to calculate the p-value and fold change in the DESeq2 software [30]. The false discovery rate (FDR) was controlled using the Benjamini-Hochberg procedure to adjust for multiple testing. *BtUGT* transcripts with an adjusted P-value < 0.05 and a fold change ≥ 2 were subsequently selected for validation by RT-qPCR.

### mRNA expression of UGTs measured by RT-qPCR

To validate the UGT expression in resistant strains, RT-qPCR was conducted focusing on five UGT genes (*BtUGT352A1*, *BtUGT352B2*, *BtUGT352B3*, *BtUGT352F1*, and *BtUGT361A1*), which showed more than 2-fold upregulation in RNA-Seq data. Approximately 50 *B. tabaci* adults of the SG20 strain were collected to extract total RNA by using TRIzol reagent (Thermo Fisher Scientific, CA, USA). Then cDNA was synthetized from 1000 ng of total RNA using an Evo *M−MLV* RT mix kit with gDNA clean for qPCR (Accurate Biology, Changsha, China). The 20 μL PCR reaction consisted of 10 μL of SYBR® Green Premix (Accurate Biology, Changsha, China), 6 μL of water, 1 μL of forward primer, 1 μL of reverse primer, and 1 μL of cDNA. The primer information was listed in Table S2. The qPCR was performed using a JENA qTOWER 3.0 real-time PCR thermal cycler (Analytik Jena, Germany), with an initial denaturation at 95 °C for 30 s, followed by 40 cycles of amplification consisting of 5 s at 95 °C and 30 s at 60 °C. According to previous studies [22,31,32], succinate dehydrogenase complex subunit A (*SDHA*) and heat shock protein 40 (*HSP40*) were chosen as reference genes. Both genes demonstrated high stability across different strains in the present study, with HSP40 Ct values having standard deviations (SD) ranging from 0.11 to 0.66 and coefficient of variation (CV) values between 0.70 % and 3.19 %. For SDHA, SD values ranged from 0.17 to 0.53, with CV values between 0.90 % and 2.79 %. Three biological replicates and three technical replicates were performed in the study. The 2^−ΔΔCt^ method was employed to quantify the relative expression levels of UGT genes.

### Function study of UGTs

The role of UGTs, specifically *BtUGT352B2* and *BtUGT352F1*, in conferring resistance to CYA, was further investigated through RNAi. Based on the results of synergist experiments and UGT enzyme assays, UGT showed a more essential role in the resistance of the SG20 strain to CYA, therefore the two genes (*BtUGT352B2* and *BtUGT352F1*) were knocked down in the SG20 strain. To minimize the possibility of non-specific silencing, the dsRNA sequence was validated through a BLASTN analysis against the *B. tabaci* genome, confirming no continuous sequence similarity of >19 nucleotides with any other transcripts. Fragments of 413 bp of *BtUGT352B2*, 370 bp of *BtUGT352F1*, and 288 bp of green fluorescent protein (GFP) were amplified with ApexHF HS DNA polymerase (Accurate Biology, China). The PCR products, after purification, were utilized for double-stranded RNA (dsRNA) synthesis following the protocol of the TranscriptAid T7 High Yield Transcription Kit (Thermo Scientific, USA). Based on previous studies [22,31,32], *B. tabaci* adults were fed a 20 % sucrose solution with 250 ng/μL dsRNA for 72 h to determine the RNAi efficiency by using RT-qPCR. To comprehensively understand the roles of two *UGT* genes in resistance, individual RNAi and combined RNAi were performed on each gene. In the treatment of combined RNAi, the dsRNA concentration for each UGT was 250 ng/μL, and dsRNA concentration of the corresponding control was elevated to 500 ng/μL.

After successful knockdown of the UGT genes, UGT enzymatic activity was measured as previously described, and the toxicity of CYA was evaluated by using a standard leaf-dip bioassay. Three biological replicates were conducted for each treatment.

### Generation of D. Melanogaster lines with overexpression of BtUGT

Total RNA was extracted from the SG20 population of *B. tabaci* using TRIzol reagent (Thermo Fisher Scientific, California, USA). First-strand cDNA synthesis was performed using the PrimeScript Ⅱ 1st Strand cDNA Synthesis Kit (Takara Biotechnology, Kyoto, Japan). The full-length coding sequences (CDS) of *BtUGT352F1* and *BtUGT352B2* were amplified in a 50 μL PCR reaction containing 25 μL ApexHF HS DNA Polymerase (Accurate Biology, Changsha, China), 2 μL of cDNA template, 1 μL of each primer (listed in Table S3), and 21 μL of nuclease-free water. The PCR conditions were as follows: an initial denaturation at 94 °C for 1 min, followed by 35 cycles of 94 °C for 15 s, 55 °C for 15 s, and 72 °C for 15 s. The amplified *BtUGT352F1* and *BtUGT352B2* were subsequently cloned into the pJFRC28-10XUAS-IVS-GFP-p10 vector (Addgene 36431) using the Uniclone One Step Seamless Cloning Kit (Genesand, Beijing, China). The resulting plasmids were transformed into DH5α competent cells, and plasmid DNA was extracted using the QIAGEN Plasmid Midi Kit (Hilden, Germany) following sequencing verification.

Each plasmid was diluted to 500 ng/μL and microinjected into embryos of the attP40 *Drosophila* line (which contains the attP site and expresses phiC31 integrase) using an Eppendorf FemtoJet 4i microinjection system. Following injection, the embryos were cultured to obtain the P0 generation. P0 adults were subsequently crossed with wild-type *Drosophila* w1118 to generate the F1 generation. Red-eyed F1 individuals were selected and crossed individually with the Bc/CyO line to obtain the F2 generation. In the F2 generation, red-eyed/CyO male and female flies were selected and self-crossed to establish the line (UAS-UGT352B2 and UAS-UGT352F1 line) which expresses UGT gene. To generate *Drosophila* overexpressing UGT genes, male UAS-UGT line was first crossed with the Sp/CyO;TM2/TM6B line to obtain the UAS-UGT/CyO;+/TM6B line. This strain was further crossed with the Sp/CyO;tub-gal4,tub-gal80ts line, resulting in the UAS-UGT/CyO;tub-gal4,tub-gal80ts/TM6B line. Finally, the UAS/CyO;tub-gal4,tub-gal80ts/TM6B line was self-crossed to establish the stable overexpression line UAS;tub-gal4,tub-gal80ts (Gal4 > UAS-UGT352B2 and Gal4 > UAS-UGT352F1 line). To overexpress the UGT genes, the Gal4 > UAS-UGT lines were subjected to a 30–32 °C heat shock for 4–6 h during the 2nd to 3rd instar larval stage. Microinjection, phenotype screening, and crossing procedures were conducted by Fungene Biotechnology Co., Ltd (Jiangsu, China).

### Toxicity assessment of *D. Melanogaster*

The toxicity of CYA to *D. melanogaster* lines was evaluated using a contact bioassay, as described in a previous study [33]. Six concentrations of CYA (10, 5, 1, 0.2, and 0.04 mg/L for the Gal4 > UAS-UGT lines, and 5, 1, 0.2, 0.04, and 0.008 mg/L for the parental lines) were prepared by dissolving the insecticide in acetone. The solutions (120 μL) were then applied to the inner surfaces of 20-mL glass scintillation vials, which were then rotated until the acetone had evaporated. A control group was included in which only 120 μL of acetone was applied. After heat shock treatment at an induction temperature of 30–32 °C for 4–6 h to activate the tub-Gal4, Gal80ts system, ten virgin female flies (3 days post-eclosion) were transferred to each vial, with three replicates per concentration. The vials were sealed with cotton balls saturated with a 5 % (w/v) glucose/water solution and incubated at 25 °C. Mortality was recorded after 48 h, and the LC_50_, slopes, 95 % confidence limit (95 % CL) values, and chi-square values were determined using PoLoPlus 2.0 (LeOra Software, California, USA).

### Expression of *BtTRE* and *BtUGPase* after exposure to CYA

To investigate the role of the UDPG biosynthesis pathway (Fig. 4A) in the resistance to CYA, two trehalase genes (*BtTRE1* and *BtTRE2*) and one UDPG pyrophosphorylase gene (*BtUGPase*) were identified based on previously published studies [34,35]. The SG20 strain was treated with the LC_50_ dose of CYA (75 mg/L) for 48 h. Following treatment, expression levels of the three genes were measured using RT-qPCR, as described above.

### Functional validation of *BtTRE2* and *BtUGPase* using RNAi

Based on the results of CYA-induced expression and the stages of enzymes in the UDPG biosynthesis pathway, *BtTRE2* and *BtUGPase* were knocked down to validate the role of the UDPG biosynthesis pathway in CYA-resistance. *B. tabaci* adults were fed on a 20 % sucrose solution containing 250 ng/μL of ds*GFP*, ds*BtTRE2*, and ds*BtUGPase*. After 48 h of feeding, the silencing efficiency of the target genes was assessed and the sensitivity of the RNAi-treated *B. tabaci* to CYA was evaluated using the leaf-dip method.

### CYA metabolite identification

To assess the involvement of UGTs in the metabolism of CYA in the resistant strain of *B. tabaci*, the potential CYA metabolites were predicted, based on the characteristics of Phase I and Ⅱ detoxification enzyme reactions, as well as previously reported CYA metabolic pathways (Fig. S2). Liquid chromatography-tandem mass spectrometry (LC-MS/MS) in product ion scan (PIS) mode was employed to detect CYA glycoside metabolites in resistant *B. tabaci* after feeding with CYA. The technical grade CYA (94 %, FMC) was dissolved in DMSO and diluted with a 20 % sucrose solution to prepare a diet containing 20 mg/L CYA. Following the feeding procedure described in section 2.7, 5000 three-day-old adult *B. tabaci* were fed the diet for 48 h, and then live adults were collected. The adult insect samples were thoroughly ground in liquid nitrogen, and 2 mL of acetone was added, followed by sonication for 30 min. The samples were then centrifuged at 3000 g for 15 min, and the supernatant was collected. Approximately 2 mL of the acetone extract was transferred to a 10 mL rotary evaporation flask and evaporated at room temperature. After evaporation, 200 µL of methanol was added to redissolve the extract, and the solution was filtered through a 0.22 µm organic filter membrane. The final extract was stored at −20 °C for subsequent analysis.

The extracts were analyzed using an AB Sciex Qtrap 5500LC/MS/MS System (AB SCIEX, Massachusetts, USA). Chromatographic separation was achieved on an Agilent Poroshell 120 EC-C18 column (2.1 × 50 mm, 1.9 µm) (Agilent Technologies, California, USA). A 2 μL aliquot of the sample was injected, and separation was achieved using a gradient elution with a mobile phase consisting of solvent A (water) and solvent B (methanol) according to the following protocol: 0–2 min, 10 % A; 2–5 min, linear increase to 50 % A; 5–10 min, increase to 80 % A; 10–12 min, increase to 95 % A; 12–16 min, increase to 100 % A. The flow rate was 0.3 mL/min, and the column temperature was maintained at 35 °C. The analysis was conducted using electrospray ionization (ESI) in positive ion mode. The experimental conditions were as follows: nebulizer gas (GS1) and curtain gas (GS2) pressures were set to 55 psi, the curtain gas (CUR) was set to 55 psi, ion source temperature (TEM) was set to 550 °C, and ion source voltage (IS) was 5500 V. In the MS1 scan, the declustering potential (DP) was set to 100 V, and the collision energy (CE) was set to 35 V. Data acquisition and analysis were performed using Analyst 1.7.3 software (AB SCIEX, Massachusetts, USA).

To identify the metabolites of CYA, a 1 mg/L CYA in standard solution was first subjected to product ion scanning (PIS) at *m*/*z* 473 to obtain characteristic fragment ion peaks. Based on the typical Phase I and Phase Ⅱ detoxification metabolic profiles in insects, as well as previously reported CYA metabolites [36], a potential metabolic network for CYA was constructed (Fig. S2). Samples were then scanned in PI mode according to the predicted *m*/*z* values of the metabolites. If the presumed Phase I and Phase Ⅱ CYA metabolites exhibited the same characteristic fragment ions as the standard compound, they were putatively identified as CYA metabolites in *B. tabaci*.

### *Homology modeling and molecular docking of* BtUGT352B2 *and* BtUGT352F1

To understand the interaction between UGT and CYA metabolites, homology modeling was conducted for BtUGT352B2 and BtUGT352F1, which have been identified as two key UGT enzymes associated with resistance in *B. tabaci*. The UGT templates with the highest similarity were selected for homology modeling by using SWISS-MODEL (https://swissmodel.expasy.org/), in which UGT708A6 (Protein Data Bank, PDB access number: 8CGQ), UGT71AP2 (PDB: 8HOJ), and UGT73P12 (PDB: 7C2X) were utilized for UGT352B2 modeling and UGT708C1 (PDB: 6LLG), UGT76G1 (PDB: 6INF) and UGT91C1(PDB: 7ERX) were used for UGT352F1 modeling. Given that the template with the highest sequence identity is below 30 %, a multi-template integration approach, followed by structural optimization, was employed to enhance the overall quality and reliability of the model. The homology models of BtUGT352B2 and BtUGT352F1 were generated using the multi-template modeling approach in MODELLER 9.23. The Phase I metabolites identified via LC-MS/MS detection were drawn using ChemDraw 20.1.1 software (PerkinElmer, Massachusetts, USA). Molecular docking analysis was conducted using AutoDock software (Scripps Research, California, USA), and the binding energies of the metabolites were calculated. The model with the lowest binding energy was selected, and the corresponding structure was saved in PDB format and imported into PyMOL (Schrödinger, New York, USA) for further visualization and structural analysis.

## Results

### Bioassay results

To investigate the potential involvement of UGTs in the resistance, synergism bioassays were conducted using two well-characterized UGT inhibitors, Sul and 5-Nul. Four resistant strains, QR, SG19, WF20 and SG20, showed 39.0-, 36.0-, 45.0-, and 33.8-fold resistance to CYA, respectively (Table 1). In QS strain, the two synergists (Sul and 5-Nul) showed little effect on the toxicity of CYA (Table 1). However, the toxicities of CYA to resistant strains were significantly increased, when two UGT inhibitors were added to CYA solutions. In the QR strain, the addition of Sul and 5-Nul increased the toxicity of CYA by 1.92 and 1.90 times, respectively. For the SG19 strain, Sul and 5-Nul produced synergistic ratios of 1.37 and 1.38 times, respectively. The WF20 strain saw synergistic ratios of 1.44 for Sul and 1.67 for 5-Nul. Most notably, in the SG20 strain, Sul and 5-Nul were particularly effective, showing synergistic ratios of 2.93 and 2.31 times, respectively. In addition, the lack of overlap in the 95 % confidence limits (CL) for SG20 strain when either Sul or 5-Nul was added indicated the significant decrease in the LC_50_ values for the strain (Table 1).

**Table 1:** Synergisms of Sulfinpyrazone (Sul), and 5-Nitrouracil (5-Nul) on cyantraniliprole (CYA) toxicity against *B. tabaci.*

Strain	Insecticide + synergist	No.^a^	Slope ± SE	LC_50_ (95 % CL ^b^) (mg L^-1^)	χ^2^(df)^c^	SR^d^	RR^e^
QS	CYA	277	1.44 ± 0.17	2.20 (1.15–4.08)	4.14 (3)	/	
	CYA + Sul	255	1.36 ± 0.19	1.88 (0.52–4.07)	4.87 (3)	1.17	
	CYA + 5-Nul	259	1.34 ± 0.20	1.76 (0.28–4.21)	6.21 (3)	1.25	

QR	CYA	241	1.21 ± 0.42	92.33 (26.61–167.98)	2.16 (3)	/	39.0
	CYA + Sul	254	1.03 ± 0.15	44.63 (30.31–69.35)	2.14 (3)	2.07	
	CYA + 5-Nul	224	1.65 ± 0.37	46.59 (22.88–71.31)	2.23 (3)	1.98	

SG19	CYA	272	1.38 ± 0.24	79.76 (53.87–118.59)	0.16 (3)	/	36.0
	CYA + Sul	276	1.24 ± 0.16	57.79 (41.10–86.09)	1.23 (3)	1.38	
	CYA + 5-Nul	276	1.41 ± 0.21	57.28 (40.08–82.05)	1.65 (3)	1.39	

WF20	CYA	280	1.45 ± 0.29	100.36 (68.56–155.20)	1.32 (3)	/	45.0
	CYA + Sul	282	1.21 ± 0.23	70.48 (43.89–110.61)	2.87 (3)	1.42	
	CYA + 5-Nul	316	1.37 ± 0.23	60.95 (40.78–87.59)	1.82 (3)	1.65	

SG20	CYA	302	1.29 ± 0.20	74.37 (51.19–111.67)	2.15 (3)	/	33.8
	CYA + Sul	280	1.36 ± 0.18	25.40 (17.49–35.67)	1.37 (3)	2.93	
	CYA + 5-Nul	265	1.46 ± 0.23	32.26 (20.15–46.99)	2.65 (3)	2.31	

^a^the number of whiteflies used in the bioassay.

^b^95 % confidence limit.

^c^Chi-square tested the linearity of the dose–mortality response. The absence of an asterisk “*” after the Chi-square value indicates the observed values are not significantly different from the expected values for the probit model (*P* > 0.05).

^d^synergism ratio = LC_50_ of CYA/ LC_50_ of CYA with synergist.

^e^resistance ratio (RR) = LC_50_ of the resistance strain without synergist/ LC_50_ of the susceptible strain without synergist.

### UGT activities in different *B. tabaci* strains

To examine the contribution of UGTs to CYA resistance, UGT activity and kinetic parameters were quantified in various *B. tabaci* strains. Compared to the susceptible QS strain (164.15 ± 30.17 pmol/min/mg protein), there was a clear increase in UGT activity in the four resistant strains. The UGT activities in QR, SG19, WF20, and SG20 increased 2.4-, 2.9-, 2.9-, and 3.7-fold, respectively (Table 2).

**Table 2:** Enzyme activity, enzymatic kinetic parameters of UGT of different strains.

Strain	UGT activity (pmol/min/mg protein)	*K_m_* ± SE (μmol/L)	95 % CL^a^ of *K_m_*	*V_max_* ± SE ^b^ (pmol/min/mg protein)	95 % CL of *V_max_*
QS	164.15 ± 30.17	14.14 ± 4.48	5.11–36.79	248.00 ± 14.14	191.50–331.00
QR	394.93 ± 26.93*	16.12 ± 1.89	11.68–22.16	598.20 ± 21.16*	545.00–658.60
SG19	478.00 ± 47.27*	17.69 ± 3.21	10.64–29.36	684.20 ± 38.32*	590.00–801.70
WF20	469.81 ± 50.79*	11.01 ± 0.87	8.94–13.52	684.70 ± 14.77*	647.40–727.40
SG20	613.51 ± 59.05*	11.54 ± 0.71	9.87–13.47	752.50 ± 12.77*	720.70–785.80

^a^95 % confidence limit.

^b^The star labeled after the data represented the significant difference between the resistant and susceptible strain.

The kinetic parameters of UGT (*K_m_* and *V_max_*) were further measured to understand the characteristics of UGT in different strains. There was no significant difference in *K_m_* values between QS and resistant strains (Table 2), which suggested UGT from different strains exhibited comparable binding affinity for the substrate α-naphthol. In contrast, *V_max_* values of resistant strains were significantly increased. The *V_max_* values in QR, SG19, SG20, and WF20 were 2.4-, 2.8-, 2.8-, and 3.0-fold of that of QS strain, respectively (Table 2), suggesting that the increased abundance of functional UGT enzymes in resistant strains provided a kinetic advantage in substrate processing capacity.

### RNA-Seq analysis for differentially expressed genes of UGTs

**Fig. 1:**
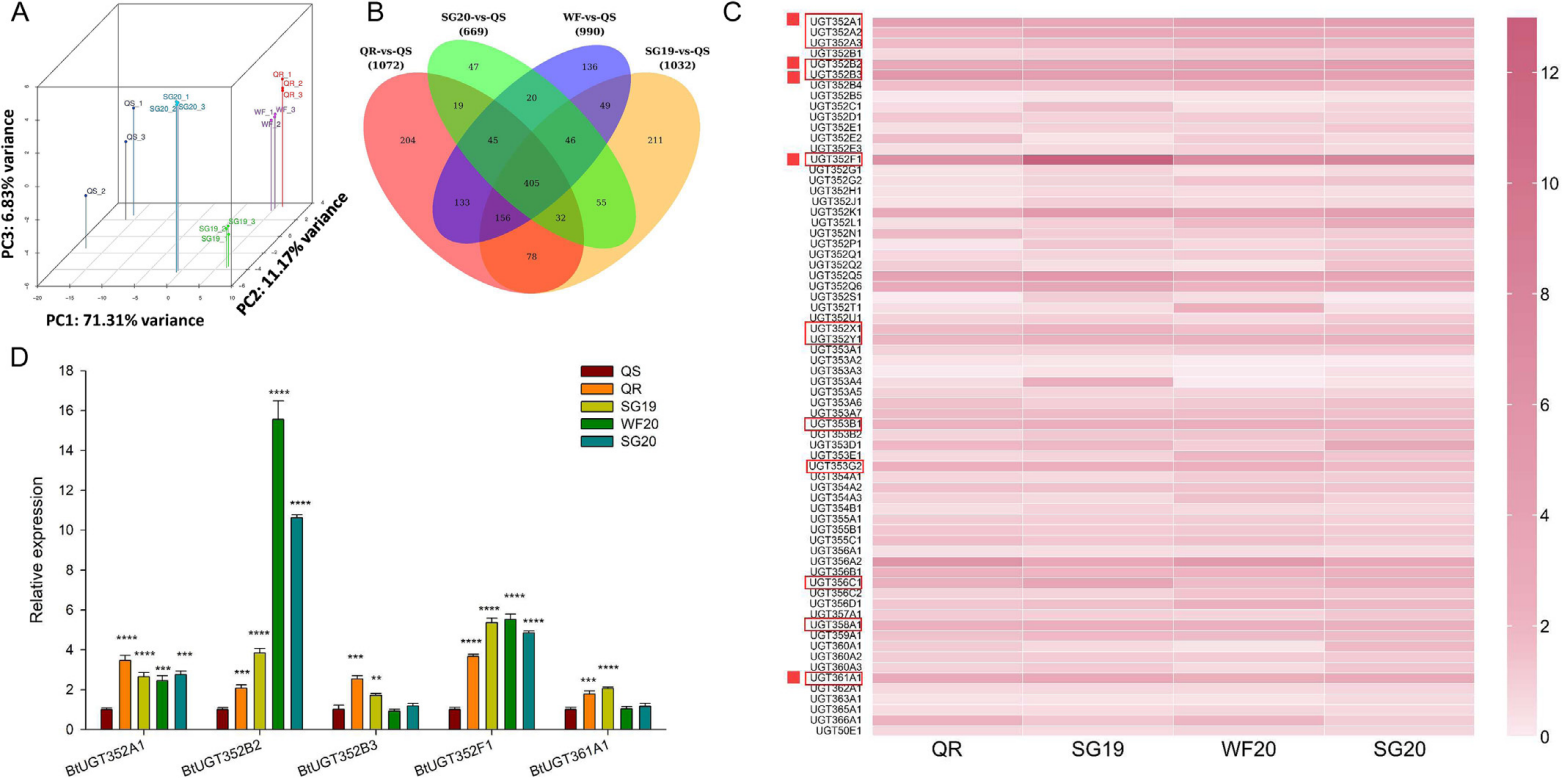
The three-dimensional principal component analysis (PCA) plot constructed using the quantitative gene expression results (A), the number of commonly and uniquely differentially expressed genes among different comparison groups (B), sixty-eight *UGT* expression profile in four resistant strains (C), and expression profiles of five UGT genes in four resistant strains, as measured by RT-qPCR (D). The heat map was generated using GraphPad Prism 7.0, in which the *UGTs* with 1.5-fold upregulation were in box, and the genes marked with small squares on the left were over 2.0-fold upregulation. Stars above the bars in panel (D) indicate significant differences between QS and the resistant strains (Student *t* test, *P < 0.05, **P < 0.01, ***P < 0.001, ****P < 0.0001).

To comprehensively identify the differentially expressed *BtUGT* genes between susceptible and resistant strains, RNA-Seq was carried out. Based on the *B. tabaci* reference genome (NCBI_ASM185493v1), a total of 12,836, 12,818, 12,814, 12,852, and 12,826 genes were identified in QS, QR, SG19, SG20, and WF20 strains, respectively, with three biological replicates per strain. The average mapping rates of each strain to the reference genome were 80.16 %, 78.80 %, 79.93 %, 79.81 %, and 79.91 %, respectively. The principal component analysis (PCA) results of gene expression levels revealed a clear separation between the susceptible strain (QS) and four resistant strains, in which principal component 1 (PC1) notably contributes to the differentiation between QS and resistant strains, accounting for 71.31 % of the total variation (Fig. 1A).

Compared with the QS strain, there were 1072, 1032, 990, and 669 differentially expressed genes in the QR, SG19, WF20, and SG20 strains, respectively. Among them, 405 differentially expressed genes were shared among the resistant strains, which are more likely to be associated with resistance to CYA (Fig. 1B, Table S1). To screen the differentially expressed genes of *BtUGT* in resistant strains, the 71 *BtUGT* genes in different strains were identified based on the previous study [37]. Due to the lack of expressions of three *BtUGT* genes (*BtUGT352C2*, *BtUGT352Q4*, and *BtUGT352W1*) in the susceptible and some resistant strains, the differentially expressed genes of 68 *BtUGTs* were analyzed in RNA-Seq results. Among the 68 *BtUGT* genes detected, 13 *BtUGTs* (*BtUGT352A1*, *BtUGT352A2*, *BtUGT352A3*, *BtUGT352B2*, *BtUGT352B3*, *BtUGT352F1*, *BtUGT352X1*, *BtUGT352Y1*, *BtUGT353B1*, *BtUGT353G2*, *BtUGT356C1*, *BtUGT358A1*, and *BtUGT361A1*) were significantly overexpressed (>1.5-fold) across all four resistant strains (*P* < 0.05). Notably, five genes (*BtUGT352A1*, *BtUGT352B2*, *BtUGT352B3*, *BtUGT352F1*, and *BtUGT361A1*) demonstrated more than 2.0-fold upregulation in these strains (Fig. 1C, Supplementary File 2).

### Expressions of five UGTs in resistant strains

To validate the upregulation of *BtUGT* genes identified by RNA-seq, RT-qPCR was employed to quantify the expression levels of five *BtUGTs* exhibiting over a 2-fold upregulation. All five *BtUGTs* significantly increased in at least two resistant strains (Fig. 1D), with three *BtUGTs*, *BtUGT352A1*, *BtUGT352B2*, and *BtUGT352F1*, showing significant increase in all four resistant strains. Notably, *BtUGT352B2* and *BtUGT352F1* exhibited markedly higher upregulation in all resistant populations. For example, *BtUGT352B2* expression increased to 2.07-, 3.84-, 15.56-, and 10.62-fold in QR, SG19, WF20, and SG20 strains, respectively. *BtUGT352F1* expression increased to 3.66-, 5.37-, 5.52-, and 4.85-fold in QR, SG19, WF20, and SG20 strains, respectively. Therefore, *BtUGT352B2* and *BtUGT352F1* were used as candidate genes to investigate in the following functional study.

### Functional study of BtUGT352B2 and BtUGT352F1 in CYA resistance

**Fig. 2:**
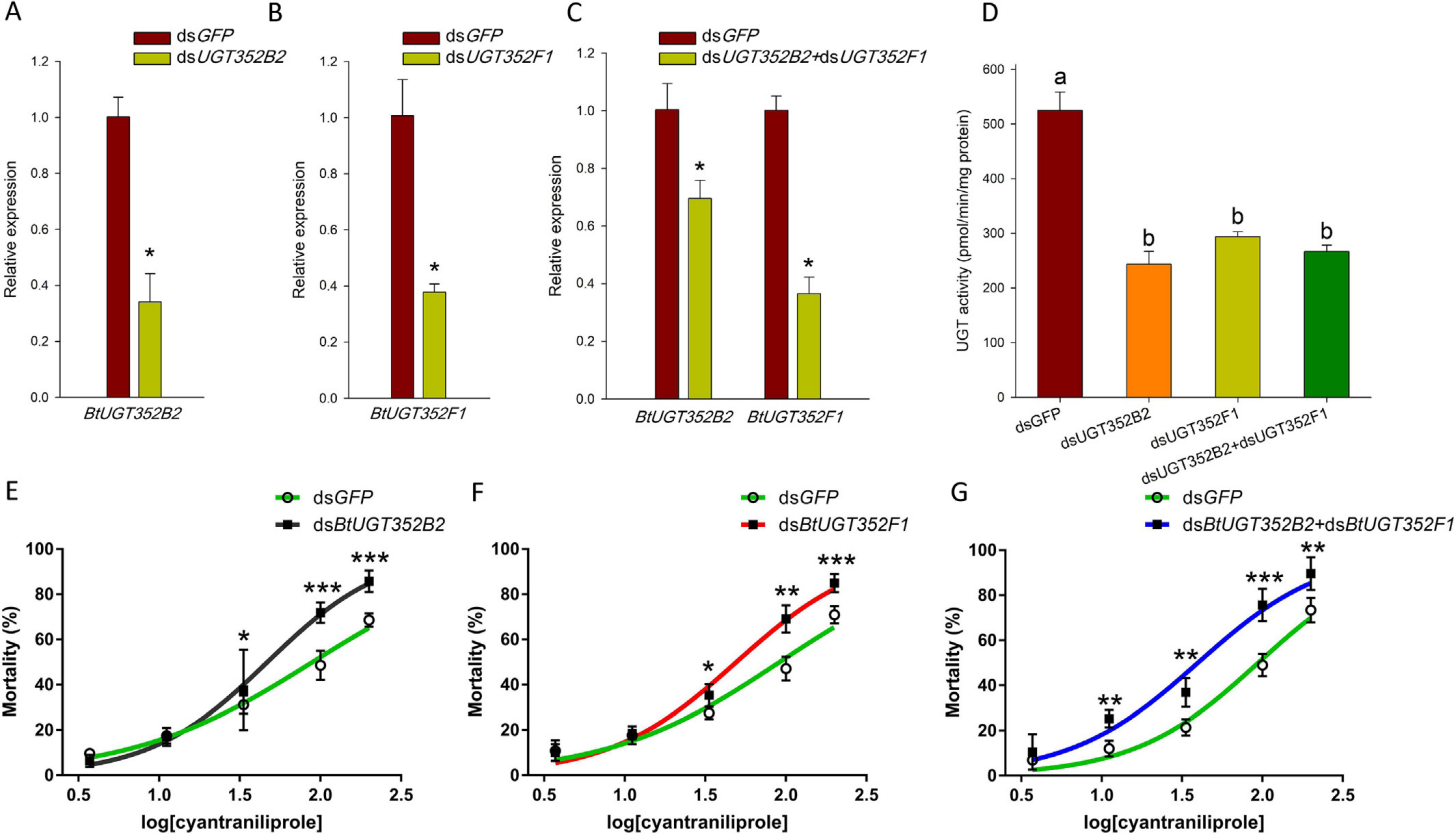
Expression levels of UGT genes in *B. tabaci* SG20 strain after feeding on different dsRNA treatments (A – C), UGT activities (D) and dose–response curves of the SG20 strain to CYA after feeding on ds*BtUGT352B2* (E), ds*BtUGT352F1* (F), and combined ds*BtUGT352B2* and ds*BtUGT352F1* (G). Data are presented as mean ± SD of three biological replicates. Bars with stars in figure B – D are significant differences (Student *t* test, *P < 0.05, **P < 0.05, ***P < 0.01). Different letters above the bars in figure D indicate significant differences (Tukey multiple comparisons test, *P* < 0.05).

To functionally validate the involvement of two candidate *BtUGT* genes in CYA resistance, RNAi assays were performed in a resistant strain. Target-specific dsRNAs were administered via artificial feeding to knock down gene expression, followed by assessments of enzymatic activity changes and alterations in insecticide resistance. After 72 h dsRNA-feeding, the expressions of target *UGT* genes significantly decreased in both individual RNAi and combined RNAi. *BtUGT352B2* expression decreased to 0.34-fold with individual ds*BtUGT352B2* feeding (Fig. 2A). *BtUGT352F1* expression decreased to 0.38-fold with individual ds*BtUGT352F1* feeding (Fig. 2B). In combined RNAi, *BtUGT352B2* and *BtUGT352F1* expressions decreased to 0.69- and 0.36-fold, respectively (Fig. 2C).

After *BtUGT352B2* and *BtUGT352F1* were successfully knocked down, UGT activities significantly decreased to 43.99–53.60 %. While the UGT activities among ds*UGT*-feeding groups showed no significant difference (Fig. 2D).

The reduced expression of these *UGT* genes significantly impacted the toxicity of CYA towards *B. tabaci*. When *BtUGT352B2* was knocked down, the mortalities of *B. tabaci* were significantly higher than control group to CYA exposure at 200.00, 100.00, and 33.33 mg/L (Fig. 2E). Knocking-down of *BtUGT352F1* also significantly increased the mortalities of *B. tabaci* to CYA treatment at the three highest concentrations (Fig. 2F). When simultaneously interfering with *BtUGT352B2* and *BtUGT352F1*, the mortalities of *B. tabaci* significantly increased at four CYA concentrations (Fig. 2G). Based on the analysis of the log dose probit response to CYA in SG20 strain after feeding on dsRNA, the LC_50_ of CYA to ds*GFP*-feeding group was 2.0-, 1.9-, and 2.6-fold higher than that of ds*BtUGT352B2*-feeding group, ds*BtUGT352F1*-feeding group, and the double interference group (Table S3).

### Toxicity of CYA against transgenic Drosophila lines

**Fig. 3:**
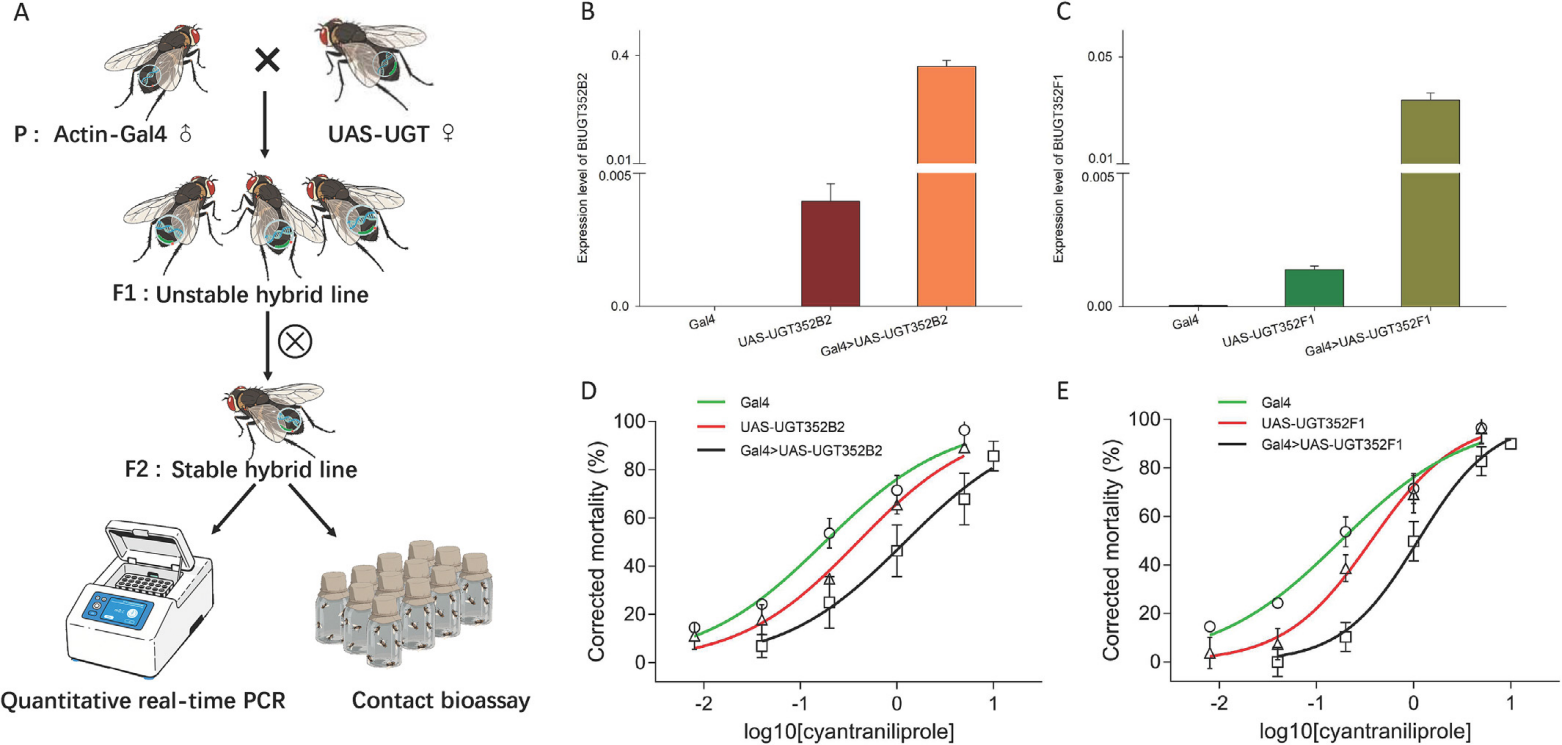
Workflow for transgenic *Drosophila* construction and phenotypic analysis (A), expression levels of *BtUGT352B2* (B) and *BtUGT352F1* (C) in different transgenic *Drosophila* lines and dose–response curves of different *Drosophila* lines to CYA (D and E).

To further confirm of the role of *BtUGT* genes in CYA resistance, transgenic *D. melanogaster* lines overexpressing *BtUGT352B2* or *BtUGT352F1* were generated using the Gal4/upstream activating sequence (UAS) system. Changes in insecticide susceptibility were then assessed through contact bioassays. Expression levels of *BtUGT352F1* and *BtUGT352B2* were first quantified in five *Drosophila* lines using RT-qPCR to evaluate the expression of the target genes in the transgenic lines. The results demonstrated that the expression level of *BtUGT352B2* in the Gal4 > UAS-UGT352B2 line was significantly higher than in both parental populations (Fig. 3A). Similarly, the expression level of *BtUGT352F1* in the Gal4 > UAS-UGT352F1 line was significantly elevated compared to the two parental populations (Fig. 3B). These findings confirmed the successful overexpression of *BtUGT352F1* and *BtUGT352B2* in the transgenic *Drosophila* lines using the GAL4/UAS system.

The toxicity of CYA to five *Drosophila* lines was evaluated using the contact bioassay method. Both Gal4 > UAS-UGT352B2 and Gal4 > UAS-UGT352F1 lines exhibited a significant increase in resistance to CYA (Fig. 3, Fig. 3). In comparison to the Gal4 control group, the LC_50_ values for the UAS-UGT352B2 line increased by 2.4-fold. For Gal4 > UAS-UGT352B2 transgenic line, the LC_50_ value increased by 6.9-fold (Table S4). Similarly, the LC_50_ values for the UAS-UGT352F1 line increased by 2.2-fold. In the case of Gal4 > UAS-UGT352F1 transgenic line, the LC_50_ value increased by 6.9-fold (Table S4).

### *BtTRE* and *BtUGPase* expression following CYA exposure

**Fig. 4:**
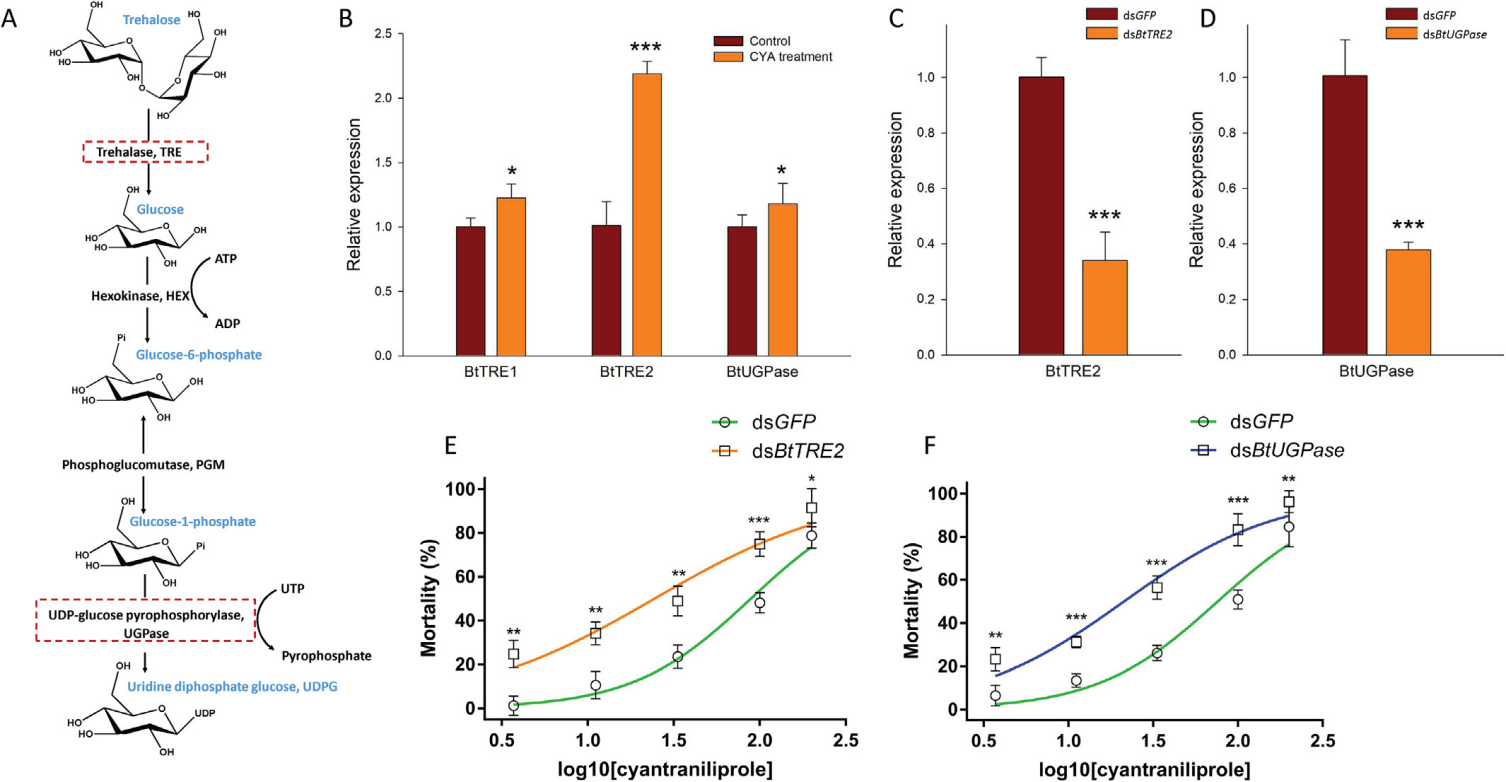
UDPG biosynthesis pathway in insects (A), expression level of the genes in the pathway after *B. tabaci* adults of SG20 strain were exposure to CYA (B), expression of *BtTRE2* and *BtUGPase* after *B. tabaci* adults of SG20 strain were fed on different dsRNA (C), and dose–response curves of SG20 strain to CYA after feeding on ds*TRE2* (D) and ds*UGPase* (E). Stars above the bars indicate significant difference between control and treatment (Student *t* test, *P < 0.05, **P < 0.01, ***P < 0.001).

To investigate the role of sugar donors in UGT-mediated detoxification, the transcriptional responses of key UDPG biosynthetic pathway genes in B. tabaci were examined following CYA exposure. CYA treatment significantly enhanced the expression of genes in the UDPG biosynthesis pathway in *B. tabaci* (Fig. 4B), and the expressions of *BtTRE1*, *BtTRE2* and *BtUGPase* were upregulated by 1.23-, 2.17- and 1.18-fold compared to the control. Given that *BtTRE2* showed high inducible expression and BtUGPase occupies a key position in the UDPG biosynthesis pathway (Fig. 4B), subsequent RNAi and functional validation studies were performed on these two genes.

### Knockdown of *BtTRE*2 and *BtUGPase* reduces CYA resistance

To functionally validate the involvement of UDPG biosynthesis in UGT-mediated detoxification in *B. tabaci*, RNAi-mediated silencing of two key genes (*BtTRE2* and *BtUGPase*) in the UDPG biosynthetic pathway was performed, followed by an assessment of changes in resistance phenotypes. Feeding on dsRNA for 72 h effectively suppressed target gene expression of *BtTRE2* and *BtUGPase* in *B. tabaci*. Compared to the control group, *BtTRE2* in the ds*BtTRE2* treatment group was significantly reduced by 33 % (Fig. 4C). Similarly, feeding ds*BtUGPase* resulted in a significant 40 % decrease in *BtUGPase* expression (*P* < 0.05) (Fig. 4D). Following successful knockdown of *BtTRE2* and *BtUGPase*, mortality rates in the ds*BtTRE2* and ds*BtUGPase* treatment groups were significantly higher at all five tested concentrations of CYA (Fig. 4, Fig. 4). LC_50_ values showed that, compared to the ds*GFP* control group (LC_50_ = 84.76 mg/L), the resistance of *B. tabaci* to CYA in the ds*BtTRE2* treatment group decreased by 3.62-fold (LC_50_ = 23.39 mg/L) (Table S5), and in the ds*BtUGPase* treatment group, resistance decreased by 4.31-fold (LC_50_ = 19.68 mg/L) (Table S6). These results suggest that interfering with key genes in the sugar donor biosynthesis pathway, likely by reducing the UDPG required for UGT metabolic reactions, can lower *B. tabaci* resistance to CYA.

### Identification of CYA metabolites in resistant strain

**Fig. 5:**
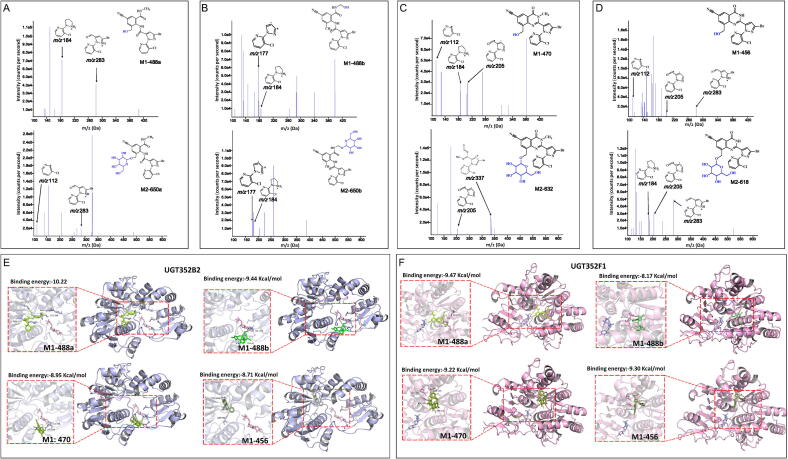
Tandem mass spectrum (MS/MS) of Phase I and II metabolites of four groups of cyantraniliprole in the *B. tabaci* (A – D), and molecular docking diagram and binding energies of BtUGT352B2 (E) and BtUGT352F1 (F) with the Phase I Metabolites of CYA. The UDP ligand is shown in light pink in Fig. 5E and light blue in Fig. 5F, and the Phase I metabolites M1-488a, M1-488b, M1-470, and M1-456 are shown in green.

To explore the metabolic pathway of CYA *in vivo*, LC-MS/MS-based metabolomic analysis was performed after resistant *B. tabaci* adults had fed on CYA for 48 h. By integrating diagnostic fragment matching with phase-specific metabolic reaction predictions, potential metabolites of CYA in *B. tabaci* were identified. LC-MS/MS analysis in PI mode of a 1 mg/L CYA standard solution identified characteristic fragment ions at a retention time of 3.411 min, including *m*/*z* 283, 205, 184, 177, 129, and 112, which served as the fingerprint for metabolite screening (Fig. S3). By combining cytochrome P450-mediated Phase I metabolic features, such as hydroxylation of saturated hydrocarbons and heterocyclic aromatics, with Phase Ⅱ glucosylation reactions catalyzed by UGTs, four sets of corresponding metabolite pairs were putatively identified: The first set includes the hydroxylated metabolite M1-488a (*m*/*z* 489) and its glucoside conjugate M2-650a (*m*/*z* 651) (Fig. 5A); the second set consists of M1-488b (*m*/*z* 489) and M2-650b (*m*/*z* 651) (Fig. 5B); the third set consists of M1-470 (*m*/*z* 471) and M2-632 (*m*/*z* 633) (Fig. 5C); and the fourth set includes M1-456 (*m*/*z* 457) and its glucoside conjugate M2-618 (*m*/*z* 619) (Fig. 5D, Supplementary File 3 and File 4).

### Binding mode of UGT with Phase I metabolites of CYA

To investigate potential interactions between UGT and Phase I metabolites of CYA, molecular docking studies were conducted using AutoDock software. Molecular docking analyses demonstrated that both BtUGT352B2 and BtUGT352F1 proteins exhibited lower binding energies with the Phase I metabolites of CYA in their native conformations (Fig. 5, Fig. 5). Specifically, the free binding energies of BtUGT352B2 with M1-488a, M1-488b, M1-470, and M1-456 were −10.22, −9.44, −8.95, and −8.71 kcal/mol, respectively, and the free binding energies of BtUGT352F1 with M1-488a, M1-488b, M1-470, and M1-456 were −9.47, −8.17, −9.22 and −9.30 kcal/mol, respectively. Metabolite M1-488a forms hydrogen bonds with key active site residues GLY-213, SER-216, LYS-366, and ARG-371 in BtUGT352B2, as well as with MET-201 and GLU-207 in BtUGT352F1. M1-488b interacts with the active pocket of BtUGT352B2 through hydrogen bonds with TRP-45 and VAL-299, and with MET-201 and GLU-207 in BtUGT352F1. M1-470 establishes hydrogen bonds with ALA-298 and VAL-299 in BtUGT352B2, and with SER-150, ASP-155, LEU-193, and ASP-381 in BtUGT352F1. M1-456 forms hydrogen bonds with ASP-80 and TYR-169 in BtUGT352B2, and with ASP-155, LEU-193, and ASP-381 in BtUGT352F1.

### UGT involvement in *B. tabaci* cross-resistance to chlorantraniliprole (CHL)

**Fig. 6:**
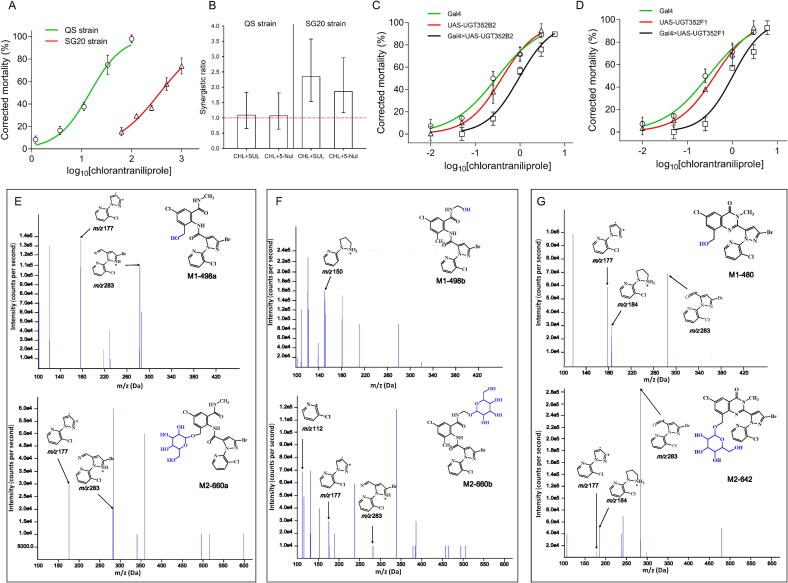
Dose-response curves of the QS and SG20 strain to chlorantraniliprole (A), synergistic ratios (SR) of sulfinpyrazone (Sul), and 5-nitrouracil (5-Nul) on chlorantraniliprole (CHL) toxicity against QS and SG20 strains (B), dose–response curves of different *Drosophila* lines to chlorantraniliprole (C and D), and tandem mass spectrum (MS/MS) of Phase I and II metabolites of three groups of chlorantraniliprole in the *B. tabaci*(E – F). In Fig. 6B, error bars indicate the 95% confidence intervals of the synergistic ratios, calculated using PoloPlus 2.0 software.

To explore the broader role of UGT-mediated metabolism in resistance to diamide insecticides, the cross-resistance of *B. tabaci* to CHL, another anthranilic diamide insecticide, was investigated, in which the cyano group (CN) in the benzene ring is replaced by a chlorine atom (Cl). There was a pronounced rightward shift in the CHL dose–response curve for SG20, indicating a substantial increase in resistance of the SG20 strain compared to the susceptible strain (Fig. 6A). Bioassay results revealed that the LC_50_ value for SG20 (398.99 mg/L, with 95 % CI of 273.03–593.13) was 27.2-fold higher than that of the QS strain (14.69 mg/L, with 95 % CI of 3.65–24.95) (Table S7). Notably, the 95 % CL for the LC_50_ values of the QS and SG20 strains did not overlap, suggesting that the SG20 strain exhibits significant cross-resistance to CHL.

To investigate the role of UGT in the metabolic resistance of *B. tabaci* to CHL, synergist bioassays were conducted. The results showed that the UGT inhibitors, Sul and 5-Nul enhanced CHL toxicity in the SG20 strain, with synergistic ratios of 2.34 and 1.86, respectively. In contrast, no significant synergistic effect was observed in the QS strain, with synergitic ratios of 1.09 and 1.07 (Fig. 6B, Table S8). These findings suggested that UGT played a role in the cross-resistance to CHL in *B. tabaci*.

To further validate the involvement of specific UGT genes, transgenic *D. melanogaster* lines overexpressing *BtUGT352B2* or *BtUGT352F1* were tested for chlorantraniliprole sensitivity. Results showed that the LC_50_ values of Gal4 > UAS-UGT352B2 line increased by 3.3-fold, compared to the control Gal4 line (Fig. 6C, Table S9). There was no overlap of the 95 % confidence limits between the Gal4 > UAS-UGT352B2 line and either UAS-UGT352B2 or Gal4 lines, suggesting that the Gal4 > UAS-UGT352B2 line was more resistance to chlorantraniliprole than the other two lines. Similarly, the Gal4 > UAS-UGT352F1 line showed higher resistance to chlorantraniliprole than the Gal4 and UAS-UGT352F1 lines, with a 3.6-fold increase in LC_50_ compared to the Gal4 line (Fig. 6D, Table S9).

After feeding the resistant strain (SG20) with a 50 mg/L chlorantraniliprole sucrose solution for 48 h, LC-MS/MS analysis was performed to identify chlorantraniliprole metabolites *in vivo*. The results putatively identified three pairs of Phase I and Phase Ⅱ metabolites, namely: M1-497a (*m*/*z* 498) and M2-659a (*m*/*z* 660), M1-497b (*m*/*z* 498) and M2-659b (*m*/*z* 660), and M1-479 (*m*/*z* 480) and M2-641 (*m*/*z* 642) (Fig. 6, Fig. 6, Supplementary File 5 and File 6). These findings provide strong evidence that UGTs contribute to cross-resistance in *B. tabaci* to chlorantraniliprole.

## Discussion

Insects can develop resistance to insecticides through both qualitative and quantitative changes in detoxification enzymes, reflecting adaptive molecular responses to chemical exposure [38]. Qualitative changes involve alternations in enzyme conformation or structure, typically caused by mutations in detoxification enzyme genes. These mutations can enhance substrate affinity or catalytic rates [39]. Quantitative changes involve adjustments in the expression levels of detoxification enzymes through transcriptional and post-transcriptional regulation, representing another pivotal mechanism in pest metabolic resistance [32,40]. To preliminarily distinguish between qualitative or quantitative changes of UGT enzymes among resistant strains, enzymatic kinetic parameters (*K_m_* and *V_max_*) were compared [41]. There was no significant variation of *K_m_* values between susceptible and resistant strains, suggesting there was no qualitative change in resistant strains. However, the *V_max_* values of resistant strains were 2.4- to 3.0-fold higher than that of the susceptible strain. Further, the increase in *V_max_* among different strains aligned with the trend in enzyme activities. The results demonstrate that the resistant *B. tabaci* strains in this study developed resistance to cyantraniliprole (CYA) through upregulation of *BtUGT* expression, thereby enhancing enzyme activity.

UGTs, as key Phase Ⅱ detoxification enzymes, play a central role in conferring resistance to diamide insecticides. For example, overexpression of *UGT33AA4* in *P. xylostella*, *UGT40AL1* and *UGT33AG3* in *C. suppressalis*, and *UGT34A23* in *T. absoluta* has been linked to resistance to chlorantraniliprole [[15], [16], [17]]. Similarly, the overexpression of multiple UGT genes (*UGT341A4*, *UGT344B4*, and *UGT344M2*) in *Aphis gossypii* were associated with the resistance to CYA [42]. To investigate how UGTs have adapted across different species and insecticide resistance, the evolutionary analysis of insect *UGTs* involved in insecticide resistance was conducted (Fig. S1). The results showed that *UGTs* belonging to three *UGT* gene families (*UGT33*, *UGT40*, and *UGT344* family) were involved in resistance to multiple insecticides and clustered together (Fig. S1). Most *UGT* genes clustered by species rather than insecticides they metabolized. For example, the *UGT* genes associated with the resistance to diamide insecticides were from different *UGT* families (*UGT33*, *UGT34*, *UGT40*, *UGT344*, *UGT341*, and *UGT352*) (Fig. S1), indicating that similar metabolic capabilities to detoxify these insecticides have evolved convergently in different *UGT* lineages. In other words, *UGTs* in different species have evolved metabolic activities as a result of their exposure to specific plants but not insecticides during the evolutionary process.

In the context of resistance to diamide insecticides, the role of detoxification enzymes, particularly cytochrome P450s and UGTs, has been demonstrated to be multifaceted and collective across various pest species [43]. On one hand, detoxification enzymes of the same class may exhibit additive effects in the metabolic degradation of diamide insecticides. In other words, the contributions of these genes to resistance are independent, with no single dominant gene. For example, four overexpressed P450 genes (*CYP6CV5*, *CYP9A68*, *CYP321F3*, and *CYP324A12*) exhibited similar effects on chlorantraniliprole resistance in *C. suppressalis* [44]. Also, the upregulation of three P450 genes (*CYP321A8*, *CYP321A9*, and *CYP321B1*) led to comparable impacts chlorantraniliprole resistance in *Spodoptera frugiperda* [45]. In the present study, there was no significant difference in the toxicity of CYA after knocking down *BtUGT352B2* and *BtUGT352F1*. Additionally, double interference of *BtUGT352B2* and *BtUGT352F1* resulted in an additive increase in mortality, suggesting that both *BtUGT352B2* and *BtUGT352F1* contributed significantly to CYA resistance in a relatively independent manner.

On the other hand, synergistic interactions between different detoxification enzyme systems (e.g., Phase I and Phase II enzymes) can collectively facilitate the metabolism of diamide insecticides. For example, resistance to CHL in a *P. xylostella* strain has been linked to the upregulation of *CYP6BG1* and *UGT2B17* [15,46]. Similarly, overexpression of P450 genes (*CYP380C6* and *CYP4CJ1*) and UGT genes (*UGT341A4*, *UGT344B4*, and *UGT344M2*) in *A. gossypii* was associated with increased resistance to CYA [42]. In the current study, synergist bioassay revealed that addition of PBO enhanced the toxicity of CYA by a factor of 1.82-fold in the SG20 strain, an effect comparable to that observed with the UGT inhibitors SUL and 5-Nul. Notably, the combined application of PBO and SUL produced a synergistic interaction, increasing efficacy by a factor of 3.26. Moreover, gene expression analysis showed significant upregulation of detoxification-related *BtP450* and *BtUGT* genes in resistant strains, with 17 % (21/126) of P450 genes and 19 % (13/68) of UGT genes being significantly overexpressed. Correspondingly, enzyme activity assays indicated a 1.71-fold increase in P450 activity and a 2.8-fold increase in UGT activity in the SG20 strain. These findings strongly suggest that both Phase I metabolism mediated by P450 enzymes and Phase II metabolism mediated by UGT enzymes are integral and closely coordinated in the metabolism of CYA. Notably, previous reports have demonstrated that the *in vitro*-expressed CYP6CX3 protein is capable of metabolizing cyantraniliprole to yield a metabolite with a mass-to-charge ratio (*m*/*z*) of 487.99993 [22], which closely corresponds to a potential Phase I metabolite (M1-488, *m*/*z* = 489) identified in this study. Furthermore, all eight CYA metabolites putatively identified here were derived from hydroxylated forms of CYA. Taken together, these results underscore the critical and interconnected roles of both Phase I and Phase II enzymatic processes in CYA metabolism, with the primary distinction lying in the sequential progression of these metabolic phases.

This study has several limitations, some of which will be addressed in future work. First, while the focus has been on mRNA levels and enzyme activity, protein-level validation, such as western blotting, is essential to confirm RNAi effects and should be included in future studies. Second, current limitations in methods for quantifying UDPG levels and consumption, combined with a limited sample size, preclude direct measurement of UDPG, a key factor in elucidating the mechanistic link between UDPG biosynthesis and UGT-mediated resistance. Moreover, the lack of reference standards for CYA metabolites limits a clear understanding of the role of UGTs in CYA’s metabolism. Future experiments will involve synthesizing phase I metabolites of CYA, enabling a more detailed analysis of the relationship between BtUGT enzyme kinetics and CYA metabolism. Additionally, subsequent studies will systematically investigate the role of *P450* genes in *B. tabaci* resistance to CYA, with the goal of providing a comprehensive understanding of the contributions of specific P450 and UGT genes to CYA resistance. Finally, this study does not consider potential fitness costs in resistant strains or the implications for virus transmission. Future research will explore the ecological role of these factors in the evolution of resistance.

## Conclusion

In summary, increased expression of UGT represents a key metabolic resistance mechanism of *B. tabaci* to CYA. Specifically, the overexpression of *BtUGT352B2* and *BtUGT352F1,* along with activation of the UDPG biosynthesis pathway exhibited a strong association with resistance. These findings improve our understanding of the involvement of UGT in the resistance to diamide insecticides and provide valuable information on the development of more sustainable pest management strategies to combat insecticide resistance.

## Declaration of competing interest

The authors declare that they have no known competing financial interests or personal relationships that could have appeared to influence the work reported in this paper.
