# Supplementary material for: UDP-glycosyltransferase confers anthranilic diamide resistance in Bemisia tabaci
Source: J Adv Res. 2025 Nov 2;86:1–13. doi: 10.1016/j.jare.2025.10.077 (PMC13453716; doi:10.1016/j.jare.2025.10.077)
Supplement: Supplementary file 1 [file mmc1.docx]

**Supporting information**

**Table S1** Number of differentially expressed genes in resistant strains

| Strain | Upregulation | Downregulation | Total |
| --- | --- | --- | --- |
| QR | 430 | 642 | 1072 |
| WF20 | 443 | 547 | 990 |
| SG20 | 320 | 349 | 669 |
| SG19 | 460 | 572 | 1032 |

**Table S2 Primers used for cDNA amplification and RT-qPCR**

| **RT-qPCR primer** | | | |
| --- | --- | --- | --- |
| **No.** | **name** | **Primer sequence** | **Amplicon (bp)** |
| 1 | BtUGT352A1-F | **AGTGCCCATTTACCTCGTGA** | 137 |
|  | BtUGT352A1-R | GGCTGTCCATTCATGCTGTT |  |
| 2 | BtUGT352B2-F | ATGCTGTACCGGGACTTTCA | 138 |
|  | BtUGT352B2-R | GTCGTCTGTATCTCCCTCCG |  |
| 3 | BtUGT352B3-F | CCAATGTCAGTCTGGCGATG | 113 |
|  | BtUGT352B3-R | GTGCAGGGGTTTTGAGATGG |  |
| 4 | BtUGT352F1-F | AAGTTCGACGTGGCCATCAT | 121 |
|  | BtUGT352F1-R | TCAAGGGGATGGTGCTGTTG |  |
| 5 | BtUGT352K1-F | CCGTATAGAGGCTGCCGATC | 103 |
|  | BtUGT352K1-R | CTGGTCCAAGCCTCGAGAAG |  |
| 6 | BtUGT352Q5-F | GCAAAGGGAGGACGACAAGA | 142 |
|  | BtUGT352Q5-R | ACGGCTGGCTTTGTAACTGA |  |
| 7 | BtUGT352Q6-F | GGGAGGGCTTCAGAGTTTCC | 147 |
|  | BtUGT352Q6-R | GAGTGGATATCTTGCGGCGA |  |
| 8 | BtUGT356A2-F | TTCGACGTCATCATAGCGCA | 108 |
|  | BtUGT356A2-R | ATCAATGGTGTGACGGCGAT |  |
| 9 | BtUGT361A1-F | GCTCGGACGATTCTCTCTGG | 80 |
|  | BtUGT361A1-R | TCTTCTGAGCAGCTGGTTGG |  |
| 10 | BtTRE1-F | ATGCACTCCCTCCTCTACGT | 101 |
|  | BtTRE1-R | AGCAAGCCTTTCACGATCCA |  |
| 11 | BtTRE2-F | ACTTGGGTGGCATTCCTACG | 112 |
|  | BtTRE2-R | TCACCAGTAGCATCCAGGGA |  |
| 12 | BtUGPase-F | GACAGAAGATGCCCGGAACA | 96 |
|  | BtUGPase-R | ACAGATTTTGGACCCCTGCA |  |
| 13 | BtHsp40-F | AGATGAGGCTCATGATGGTCAA | 51 |
|  | BtHsp40-R | TGAGAAGCGCATTGCATTGT |  |
| 14 | BtSDHA-F | GCGACTGATTCTTCTCCTGC | 141 |
|  | BtSDHA-R | TGGTGCCAACAGATTAGGTGC |  |
| 15 | DmRPL11-F | CGATCCCTCCATCGGTATCT | 141 |
|  | DmRPL11-R | AACCACTTCATGGCATCCTC |  |
| **dsRNA primer** | | | |
| 12 | dsBtUGT352B2-F | taatacgactcactatagggCTCGGAGGGAGATACAGACG | 413 |
|  | dsBtUGT352B2-R | taatacgactcactatagggTGAATCCGGAAACGAGGTAG |  |
| 13 | dsBtUGT352F1-F | taatacgactcactatagggGGCCATCATAGAATTCGTGG | 370 |
|  | dsBtUGT352F1-R | taatacgactcactatagggCGCATTGGACGATATCATTG |  |
| 14 | dsGFP-F | taatacgactcactatagggCAGTGCTTCAGCCGCTAC | 288 |
|  | dsGFP-R | taatacgactcactatagggGTTCACCTTGATGCCGTTC |  |
| 15 | dsBtTRE2-F | taatacgactcactatagggGAGTACGGACTCATCCCGAA | 547 |
|  | dsBtTRE2-R | taatacgactcactatagggCCTCCATCCAATCCTTGCTA |  |
| 16 | dsBtUGPase-F | taatacgactcactatagggGCAGGGGTCCAAAATCTGTA | 485 |
|  | dsBtUGPase-R | taatacgactcactatagggCCTGGTTTTGTCGGTGACTT |  |
| **Primers for transgenic *Drosophila melanogaster* lines** | | | |
| 15 | UAS-BtUGT352B2-F | TCTTATCCTTTACTTCAGGCGGCCGCAAAATGATTTTCGCAAAATGCTATC | 1629 |
|  | UAS-BtUGT352B2-R | GTTATTTTAAAAACGATTCATTCTAGATTATTTATTTTTGTGCGAAAATGAACC |  |
| 16 | UAS-BtUGT352F1-F | TCTTATCCTTTACTTCAGGCGGCCGCAAAATGGCTTCGTCTCTGAAACT | 1599 |
|  | UAS-BtUGT352F1-R | GTTATTTTAAAAACGATTCATTCTAGATCACGACTTTTTTTCTTTAGAACTG |  |

**Table S3** Log dose-probit response of SG20 strain to CYA after feeding on dsRNA

|  | No.^a^ | Slope±SE | LC_50_ (95%CL ^a^) (mg/L) | χ^2^(df) |
| --- | --- | --- | --- | --- |
| ds*GFP* | 330 | 1.06 ± 0.16 | 89.78 (60.78 – 186.25) | 0.59 (3) |
| ds*UGT352B2* | 335 | 1.59 ± 0.20 | 45.73 (33.69 – 60.39) | 0.89 (3) |
| ds*GFP* | 361 | 1.02 ± 0.16 | 88.63 (41.75 – 279.11) | 3.54 (3) |
| ds*UGT352F1* | 327 | 1.47 ± 0.25 | 47.84 (29.39 – 68.52) | 2.20 (3) |
| ds*GFP* | 351 | 1.60 ± 0.46 | 100.04 (60.17 – 144.18) | 2.48 (3) |
| ds*UGT352B2* + ds*UGT352F1* | 368 | 1.55 ± 0.25 | 38.91 (23.50 – 54.95) | 2.78 (3) |

a, the number of whiteflies used in the bioassay.

b, 95% confidence limit

**Table S4** Log dose-probit response to CYA and resistance ratios of *Drosophila melanogaster* lines.

| Line | Number | Slope ± SE | LC50 | 95%CL ^b^ (mg/L) | χ2(df) ^c^ | RR ^d^ |
| --- | --- | --- | --- | --- | --- | --- |
| Gal4 | 180 | 0.99 ± 0.16 | 0.17 | 0.08 – 0.33 | 1.96 (3) | - |
| UAS-UGT352B2 | 177 | 1.01 ± 0.25 | 0.40 | 0.11 – 0.84 | 0.81 (3) | 2.4 |
| Gal4>UAS-UGT352B2 | 180 | 0.97 ± 0.19 | 1.18 | 0.48 – 2.33 | 0.74 (3) | 6.9 |
| UAS-UGT352F1 | 178 | 1.45 ± 0.31 | 0.37 | 0.16 – 0.66 | 0.55 (3) | 2.2 |
| Gal4>UAS-UGT352F1 | 180 | 1.49 ± 0.23 | 1.17 | 0.68 – 1.82 | 0.49 (3) | 6.9 |

a, the number of *Drosophila melanogaster* which was exposed to different concentrations of cyantraniliprole.

b, 95% confidence limits.

c, Chi-square tested the linearity of dose–mortality response conducted by POLOPlus.. No “*” sign after the Chi-square and degrees of freedom (df) indicates the observed values are not significantly different from the expected values for the probit model (*P* > 0.05).

d, Resistance ratio = LC_50_ of *D. melanogaster* line/LC_50_ of Gal4 line

**Table S5** Log dose-probit response of SG20 strain to CYA after feeding on dsRNA

|  | Number^a^ | Slope±SE | LC_50_ (95%CL) (mg/L) ^b^ | χ^2^(df) ^c^ |
| --- | --- | --- | --- | --- |
| ds*GFP* | 287 | 1.61± 0.42 | 82.20 (47.77 – 119.27) | 2.44 (3) |
| ds*BtTRE2* | 277 | 1.13 ± 0.18 | 23.39 (8.69 – 47.89) | 3.04 (3) |

a, The number of *B. tabaci* which was exposed to different concentrations of CYA.

b, 95% confidence limits.

c, Chi-square tested the linearity of dose–mortality response conducted by POLOPlus.. No “*” sign after the Chi-square and degrees of freedom (df) indicates the observed values are not significantly different from the expected values for the probit model (*P* > 0.05).

**Table S6** Log dose-probit response of SG20 strain to CYA after feeding on dsRNA

|  | Number^a^ | Slope±SE | LC_50_ (95%CL) (mg/L) ^b^ | χ^2^(df) ^c^ |
| --- | --- | --- | --- | --- |
| ds*GFP* | 313 | 1.74 ± 0.18 | 86.29 (57.14 – 121.86) | 1.93 (3) |
| ds*BtUGPase* | 314 | 1.48 ± 0.19 | 20.83 (8.68 – 36.91) | 3.71 (3) |

a, The number of *B. tabaci* which was exposed to different concentrations of CYA.

b, 95% confidence limits.

c, Chi-square tested the linearity of dose–mortality response conducted by POLOPlus.. No “*” sign after the Chi-square and degrees of freedom (df) indicates the observed values are not significantly different from the expected values for the probit model (*P* > 0.05).

**Table S7** Log dose-probit response of QS and SG20 strain to chlorantraniliprole

|  | Number^a^ | Slope±SE | LC_50_ (95%CL) (mg/L) ^b^ | χ^2^(df) ^c^ | RR^d^ |
| --- | --- | --- | --- | --- | --- |
| QS | 277 | 2.00 ± 0.40 | 14.69 (3.65 – 24.95) | 3.26 (3) |  |
| SG20 | 289 | 1.46 ± 0.28 | 398.99 (273.03 – 593.13) | 0.66 (3) | 27.2 |

a, The number of *B. tabaci* which was exposed to different concentrations of chlorantraniliprole.

b, 95% confidence limits.

c, Chi-square tested the linearity of dose–mortality response conducted by POLOPlus.. No “*” sign after the Chi-square and degrees of freedom (df) indicates the observed values are not significantly different from the expected values for the probit model (*P* > 0.05).

d, resistance ratio (RR) = LC_50_ of the resistance strain (SG20)/ LC_50_ of the susceptible strain (QS).

**Table S8** Synergisms of Sulfinpyrazone (Sul), and 5-Nitrouracil (5-Nul) on chlorantraniliprole (CHL) toxicity against *B. tabaci*

| Strain | Insecticide + synergist | No.^a^ | Slope±SE | LC_50_ (95% CL ^b^) (mg L^-1^) | χ^2^(df)^c^ | SR^d^ | RR^e^ |
| --- | --- | --- | --- | --- | --- | --- | --- |
| QS | CHL | 301 | 1.48 ± 0.22 | 15.92 (10.61 – 22.37) | 1.83 (3) | / |  |
|  | CHL + Sul | 289 | 1.50 ± 0.22 | 14.63 (9.62 – 20.67) | 1.72 (3) | 1.09 |  |
|  | CHL + 5-Nul | 287 | 1.55 ± 0.23 | 14.93 (9.58 – 21.24) | 0.71 (3) | 1.07 |  |
| SG20 | CHL | 280 | 1.79 ± 0.27 | 422.04 (293.41 – 568.50) | 0.43 (3) | / | 39.0 |
|  | CHL + Sul | 274 | 1.79 ± 0.19 | 180.25 (135.30 – 234.95) | 0.35 (3) | 2.34 |  |
|  | CHL + 5-Nul | 271 | 2.04 ± 0.31 | 227.01 (151.11 – 305.79) | 1.15 (3) | 1.86 |  |

a the number of whiteflies used in the bioassay.

b 95% confidence limit.

c Chi-square tested the linearity of dose–mortality response. The absence of an asterisk “*” after the Chi-square value indicates the observed values are not significantly different from the expected values for the probit model (*P* > 0.05).

d synergism ratio = LC_50_ of CYA/ LC_50_ of CYA with synergist.

e resistance ratio (RR) = LC_50_ of the resistance strain without synergist/ LC_50_ of the susceptible strain without synergist.

**Table S9** Log dose-probit response to chlorantraniliprole and resistance ratios of *Drosophila melanogaster* lines.

| Line | Nnmber ^a^ | Slope ± SE | LC_50_ | 95%CL ^b^ (mg/L) | χ2(df) ^c^ | RR ^d^ |
| --- | --- | --- | --- | --- | --- | --- |
| Gal4 | 179 | 1.20 ± 0.25 | 0.30 | 0.12 – 0.55 | 0.61 (3) | - |
| UAS-UGT352B2 | 179 | 1.54 ± 0.27 | 0.38 | 0.22 – 0.59 | 0.23 (3) | 1.3 |
| Gal4>UAS-UGT352B2 | 181 | 1.65 ± 0.27 | 0.98 | 0.62 – 1.43 | 0.81 (3) | 3.3 |
| UAS-UGT352F1 | 180 | 1.52 ± 0.31 | 0.41 | 0.21 – 0.65 | 0.63 (3) | 1.4 |
| Gal4>UAS-UGT352F1 | 180 | 1.83 ± 0.33 | 1.09 | 0.66 – 1.58 | 2.46 (3) | 3.6 |

a, the number of *Drosophila melanogaster* which was exposed to different concentrations of chlorantraniliprole.

b, 95% confidence limits.

c, Chi-square tested the linearity of dose–mortality response conducted by POLOPlus.. No “*” sign after the Chi-square and degrees of freedom (df) indicates the observed values are not significantly different from the expected values for the probit model (*P* > 0.05).

d, Resistance ratio = LC_50_ of *D. melanogaster* line/LC_50_ of Gal4 line.

**Figure S1** Phylogenetic tree of *UGT* genes of *B. tabaci* and other insect *UGT*s involved in insecticide resistance. The genes highlighted in yellow were involved in resistance to diamide insecticides. The two *UGTs* investigated in this study were marked with red points. The phylogenetic tree was constructed through Clustal W alignment of gene nucleotide sequences, employing the maximum-likelihood method in MEGA 7.0. Bootstrap values were computed from 1,000 replicates, with a cutoff set at <50%. The letters preceding the genes denote various insects, in which Bt represented *Bemisia tabaci*; Ag represented *Aphis gossypii*; Cs represented *Chilo suppressalis*; Dc represented *Diaphorina citri*; Dm represented *Drosophila melanogaster*; Fo represented *Frankliniella occidentalis*; Ha represented H*elicoverpa armigera*; Mp represented *Myzus persicae*; Nl represented *Nilaparvata lugens*; Px represented *Plutella xylostella*; Ta represented *Tuta absoluta*.

**Figure S2** Predicted Phase I and Ⅱ metabolites of CYA in *B. tabaci.* The metabolites detected by LC-MS/MS are highlighted within the red dashed box.

**Figure S3** Tandem mass spectrum (MS/MS) of cyatraniliprole (A) and chlorantraniliprole (B)
